# Supplementary material for: Common and rare genetic variants show network convergence for a majority of human traits
Source: EMBO Rep. 2026 Mar 26;27(8):1918–43. doi: 10.1038/s44319-026-00733-4 (PMC13121720; doi:10.1038/s44319-026-00733-4)
Supplement: Supplementary file 1 — Appendix [file 44319_2026_733_MOESM1_ESM.pdf]

APPENDIX

Common and rare genetic variants show network convergence for a majority of human traits

Sarah N. Wright[1], Jane Yang[1], Trey Ideker[1,2,*]

[1]Department of Medicine, University of California, San Diego, La Jolla, CA 92093, USA
[2]Institute for Genomic Medicine, University of California, San Diego, La Jolla, CA 92093, USA
*Corresponding Author

Table of Contents

Appendix Figure S1. Pathway and phenotype enrichment of prioritized glucose measurement-associated genes. 2
Appendix Figure S2. Optimization of network colocalization inputs and parameters…………………………………. 3
Appendix Figure S3. Benchmarking of network colocalization…………………………………………………………... 4

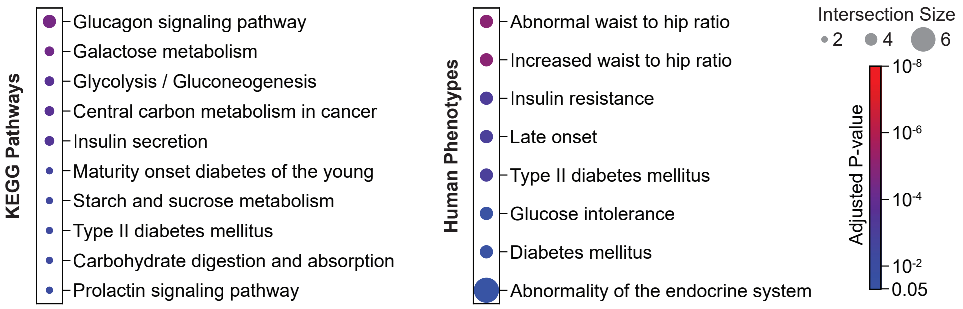

Appendix Figure S1. Pathway and phenotype enrichment of prioritized glucose measurement-associated genes. The top 10 KEGG pathway enrichments and all significant Human Phenotype (HP) ontology enrichments are shown for the ten glucose measurement trait-associated genes that were prioritized by the network analysis. Of the ten genes, six were present in the KEGG and HP databases.

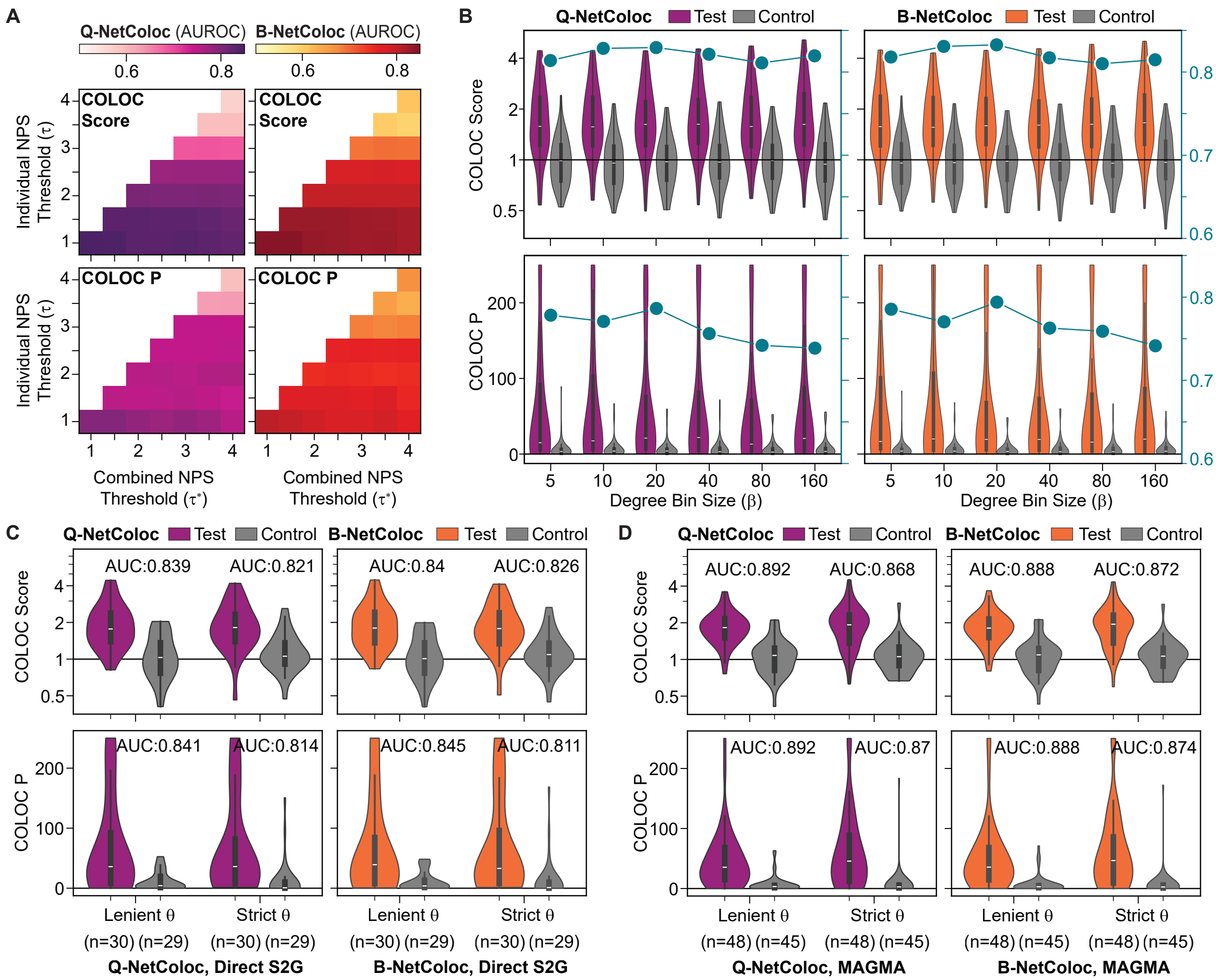

Appendix Figure S2. Optimization of network colocalization inputs and parameters. Quantitative (Q-NetColoc) and Binary (B-NetColoc) implementations are shown in purple and orange, respectively. All analyses were conducted with partitioned GWAS results for traits not included in the main analysis. Test results are from matched study partitions, while control results are from analysis of unrelated partitions (Methods). **A**) AUROC results for Q-NetColoc and B-NetColoc across different thresholds for defining the trait-specific network. **B**) Network colocalization results and AUROC values for Q-NetColoc and B-NetColoc across different bin sizes used for degree-matched randomization. **C**) Network colocalization results and AUROC values for Q-NetColoc and B-NetColoc at lenient ($p < 1\times10^{-5}$) and strict ($p < 1\times10^{-8}$) thresholds for inclusion of trait-associated genes. Trait-associated genes defined by direct SNP-to-Gene (S2G) mapping via the GWAS catalog. **D**) Network colocalization results and AUROC values for Q-NetColoc and B-NetColoc at lenient ($p < 1\times10^{-4}$) and strict ($p < 2.5\times10^{-6}$) thresholds for inclusion of trait-associated genes. Trait-associated genes defined by MAGMA gene-level p-values. For all violin plots, the center box plots show the median COLOC score or COLOC P and interquartile range (IQR), with the lower and upper whiskers extending to Q1 − 1.5IQR and Q3 + 1.5IQR. The violins extend to the minimum and maximum observations. Related to Methods and Dataset EV2.

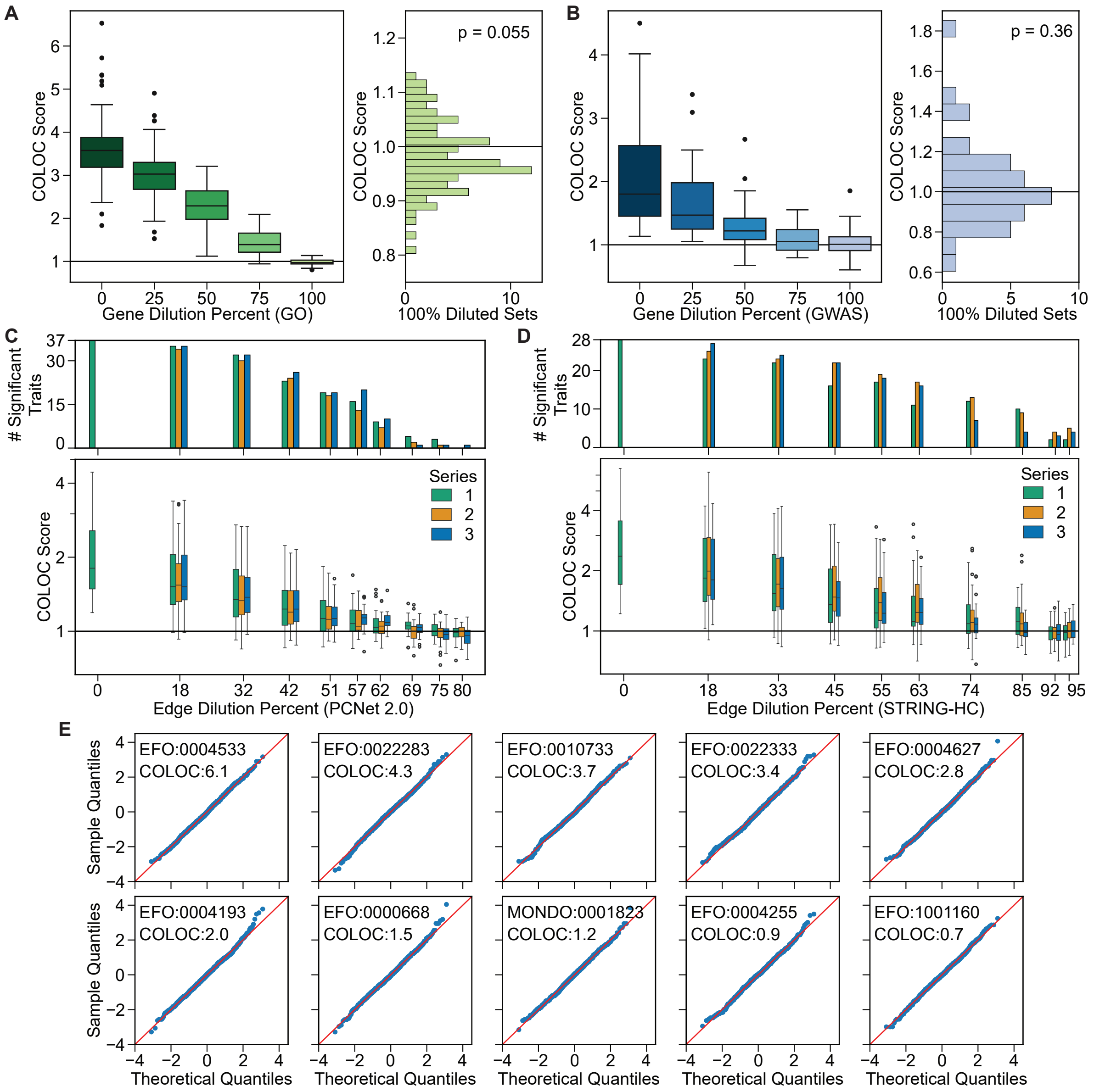

Appendix Figure S3. Benchmarking of network colocalization. **A-B**) COLOC score as a function of gene set dilution for (**A**) B-NetColoc with GO gene sets and (**B**) Q-NetColoc with partitioned GWAS gene sets. The dilution percent indicates the percent of input gene set genes replaced with degree-matched random genes. Results for GO gene sets averaged over three repeats, and results for partitioned GWAS averaged over five repeats. Box plots show the median COLOC score and interquartile range (IQR), with the lower and upper whiskers extending to Q1 − 1.5IQR and Q3 + 1.5IQR. Histograms show the distribution of COLOC score for completely randomized gene sets; p-values calculated using a 1-sample t-test with null hypothesis μCOLOC = 1. **C-D**) COLOC scores for partitioned GWAS test traits across randomized versions of (**C**) PCNet 2.0 and (**D**) STRING-HC. Test traits were filtered to those with a significant network colocalization using the original network (PCNet 2.0: n = 37, STRING-HC: n = 28). For each network, three series were generated by progressively diluting the original network (dilution = 0%) via degree-preserving edge swaps. Dilution percent is determined as 100×(1−J), where J is the Jaccard similarity of edges in the original and randomized networks. Box plots show the median COLOC score and interquartile range (IQR), with the lower and upper whiskers extending to Q1 − 1.5IQR and Q3 + 1.5IQR. Top bar plots show the number of test inputs displaying significant network colocalization (COLOC P < 0.05). **E**) Quantile-quantile plots for the expected distribution of colocalized network size for ten traits with varying COLOC score. All traits were ranked by COLOC score, with every 40th trait selected. For each trait, the expected distribution of sizes was calculated via 1000 permutations of the NPS scores and then standardized. Related to Methods and Dataset EV3.
