# Supplementary material for: Common and rare genetic variants show network convergence for a majority of human traits
Source: EMBO Rep. 2026 Mar 26;27(8):1918–43. doi: 10.1038/s44319-026-00733-4 (PMC13121720; doi:10.1038/s44319-026-00733-4)
Supplement: Supplementary file 12 — Expanded View Figures [file 44319_2026_733_MOESM12_ESM.pdf]

Expanded View Figures

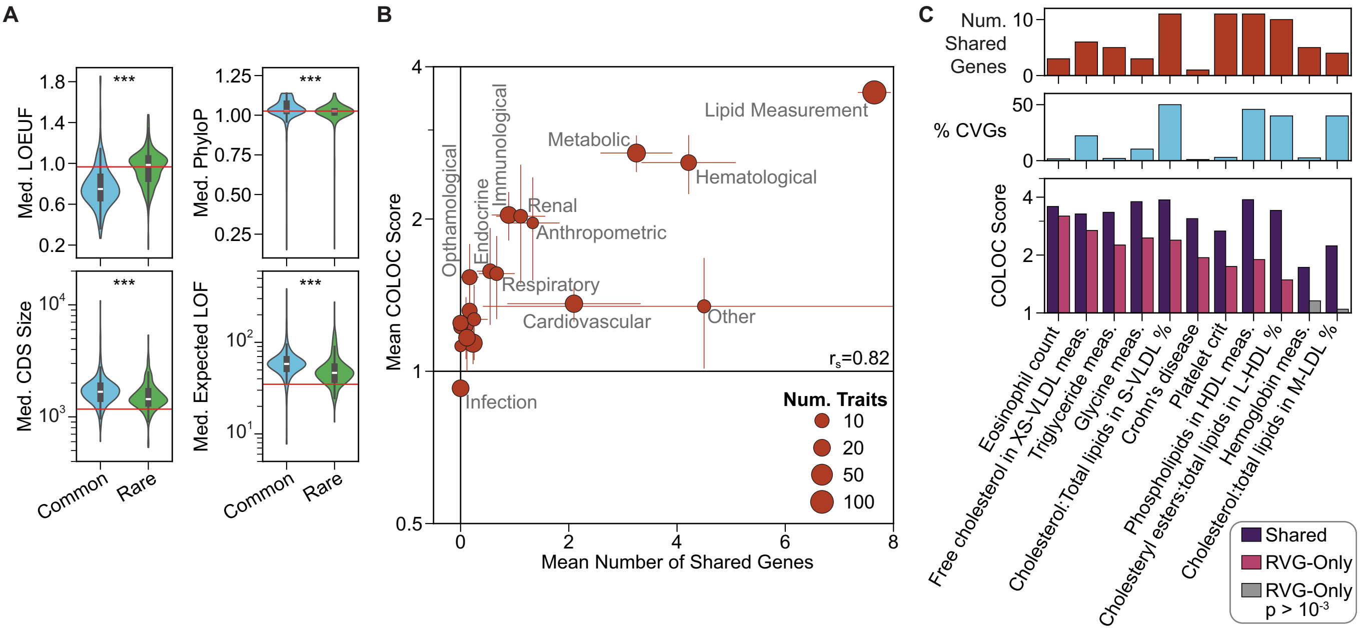

Figure EV1. Further exploration of gene properties, disease groups, and synthetic associations.

(**A**) Comparison of CVG and RVG properties across 373 traits using alternative metrics of gene constraint and gene size. The value for each set of CVGs or RVGs was taken as the median of all genes in each set (Wilcoxon Signed-Rank test, BH correction). The center box plots show the median property value and interquartile range (IQR), with the lower and upper whiskers extending to Q1 − 1.5IQR and Q3 + 1.5IQR. The violins extend to the minimum and maximum observations. Red lines indicate the median value for all protein-coding genes. ***$q < 1 \times 10^{-5}$. $q_{MedLOEUF} = 5.2 \times 10^{-34}$, $q_{MedPhyloP} = 4.2 \times 10^{-11}$, $q_{MedCDSSize} = 8.4 \times 10^{-9}$, $q_{MedExpectedLOF} = 1.4 \times 10^{-13}$. (**B**) Comparison of the COLOC score and the number of shared CVG and RVG genes for traits within each biological domain. Points represent the mean values within each domain, with error bars representing the standard errors. Point size indicates the number of traits per domain. (**C**) Estimation of the impact of synthetic associations on COLOC scores. Ten random traits with at least 3 shared genes identified by CV and RV association studies, and Crohn's disease (a trait with a known synthetic association), were analyzed by removing shared genes from the set of CVGs. The top bar plot shows the number of shared genes for each trait, and the middle bar plot shows the shared genes as a percentage of all CVGs. The bottom bar plot shows the COLOC scores for each trait, allowing shared genes or treating shared genes as RVGs only. Colored bars represent significant network colocalizations ($p < 10^{-3}$).

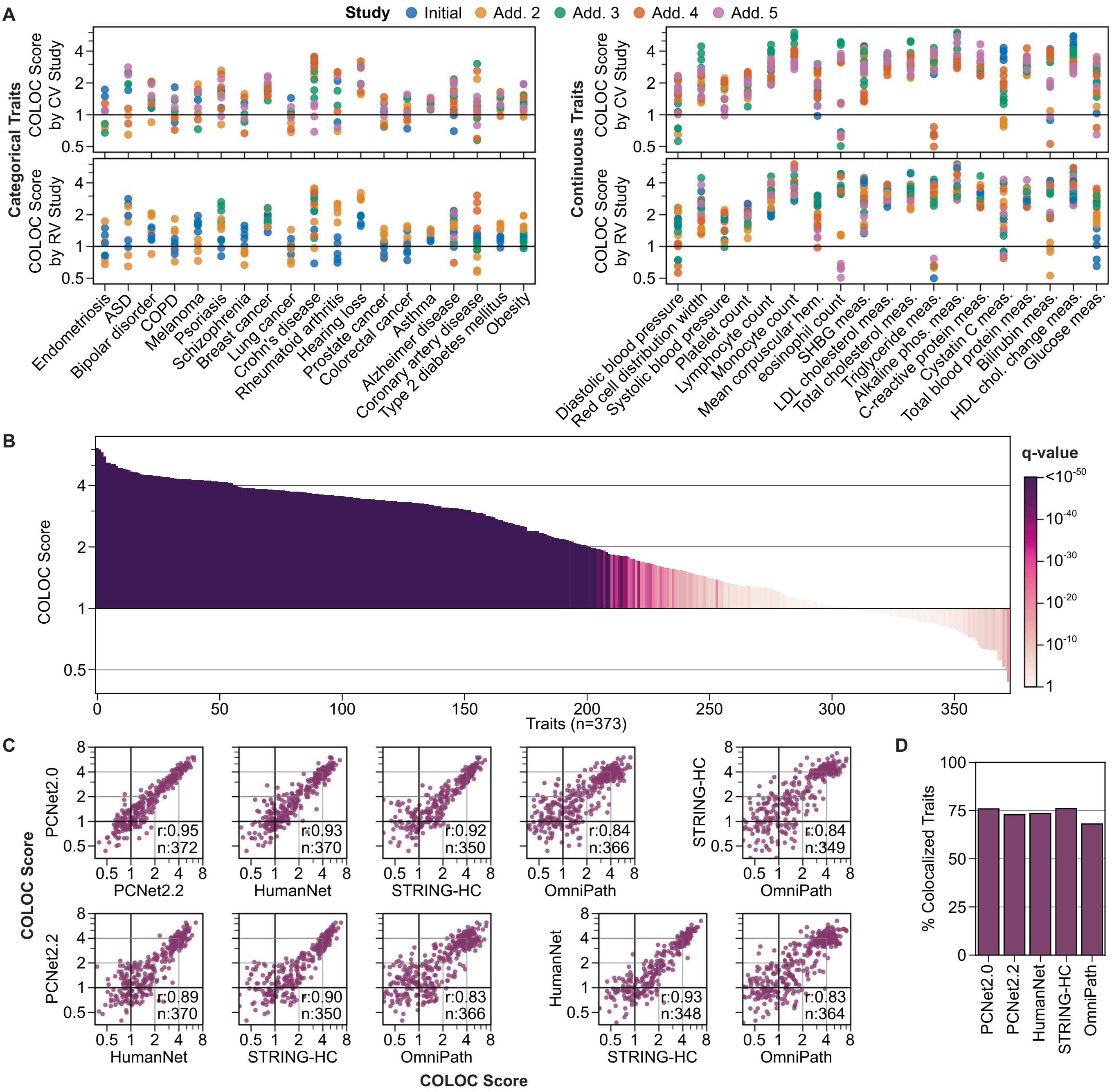

Figure EV2. Robustness of network convergence across different studies and networks.

(**A**) COLOC scores for categorical traits with ≥10 rare-common study combinations (left, $n = 19$) and continuous traits with ≥20 common-rare study combinations (right, $n = 19$). The top plots are colored by CV study, and the bottom plots are colored by RV study. ASD: autism spectrum disorder, COPD: Chronic Obstructive Pulmonary Disease. Point colors indicate whether each study was part of the initial or an additional study combination. (**B**) Network colocalization results for 373 traits after taking the highest COLOC score from all available common-rare study analyses per trait. Traits are ordered by COLOC score, and bar color indicates q-value (BH correction) of the COLOC score based on a Z-test against 1000 permutations. (**C**) Pairwise comparison of COLOC scores across five biological networks. Correlation (r) calculated using Spearman correlation. The number of traits that could be assessed by both networks is given by *n*. (**D**) For each network, the fraction of evaluated traits with a nominally significant COLOC score ($p < 0.05$). Related to Fig. 3.

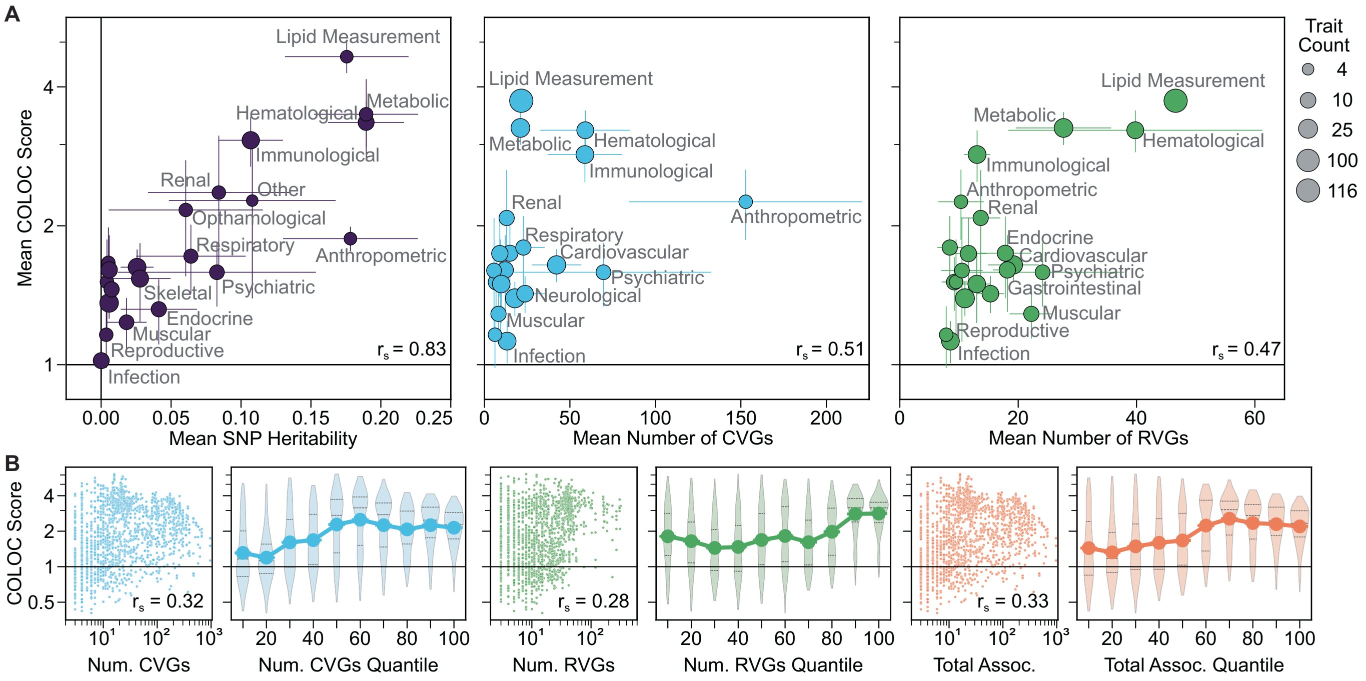

Figure EV3. Network convergence as a function of gene set and biological properties.

(**A**) Correlation of mean COLOC score across biological domains with trait SNP heritability, number of CVGs, and number of RVGs. Points represent the mean values within each domain, with error bars representing the standard errors. Point size indicates the number of traits per domain. Heritability results are shown for the subset of 197 traits for which a SNP heritability estimate was available, and the number of gene results are shown for the 373 best trait study pairs (Fig. EV2B). (**B**) COLOC score as a function of the number of underlying CVG and RVG associations for all combinations of initial and additional studies ($n = 1634$). Total association count is the total number of distinct CVGs and RVGs per trait. Spearman correlation reported. Line plots show the mean COLOC score for each quantile, and violin plots show the distribution of COLOC scores within each feature quantile, with the median and quartiles represented by horizontal lines. Violins extend to the minimum and maximum observed values.

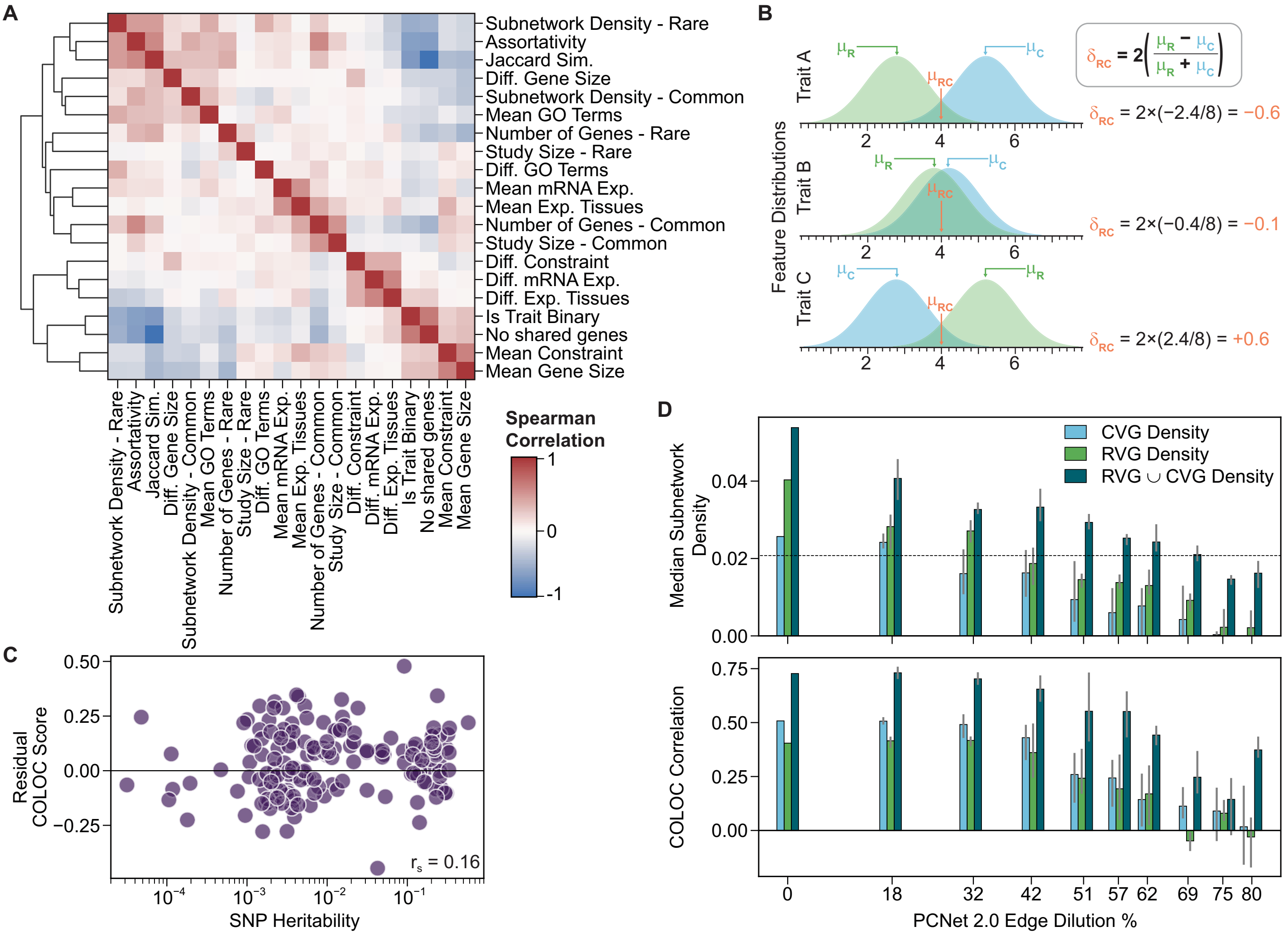

Figure EV4. Expanded analysis of gene and trait features.

(**A**) Correlation of gene and trait features over 1634 common-rare study combinations for 373 traits. Jaccard similarity was calculated between the sets of CVGs and RVGs. Gene features were summarized by the overall mean of CVGs and RVGs, and the difference between CVGs and RVGs. Constraint: gene selective constraint defined by s_{het} estimates. Exp. Tissues: Number of tissues with mRNA expression >1 TPM. (**B**) Schematic showing the calculation of mean (μ) and difference (δ) metrics for gene features. Distributions represent the hypothetical feature values for all CVGs (blue) and RVGs (green) of a given trait. Where the RVGs have higher average property values, the δ value will be positive. (**C**) Residual COLOC score as a function of estimated SNP heritability for the 197 traits with available heritability data. Residual COLOC score calculated as the difference between the observed COLOC score and the COLOC score predicted by the Elastic Net regression model following five-fold cross-validation. Spearman correlation reported. (**D**) Subnetwork density of RVGs and CVGs, and Spearman correlation with COLOC score for partitioned GWAS test traits across progressively randomized versions of PCNet 2.0. Three series of randomized networks were generated by progressively diluting PCNet 2.0 via degree-preserving edge swaps, with subnetwork density calculated with each network structure. Dilution percent is determined as 100 × (1 − J), where J is the Jaccard similarity of edges in the original and randomized networks. Bar plots show the values for the original network (dilution = 0%, *n* = 1) or the median value for the diluted networks (dilution > 0%, *n* = 3), with error bars indicating the minimum and maximum values. Related to Fig. 4.

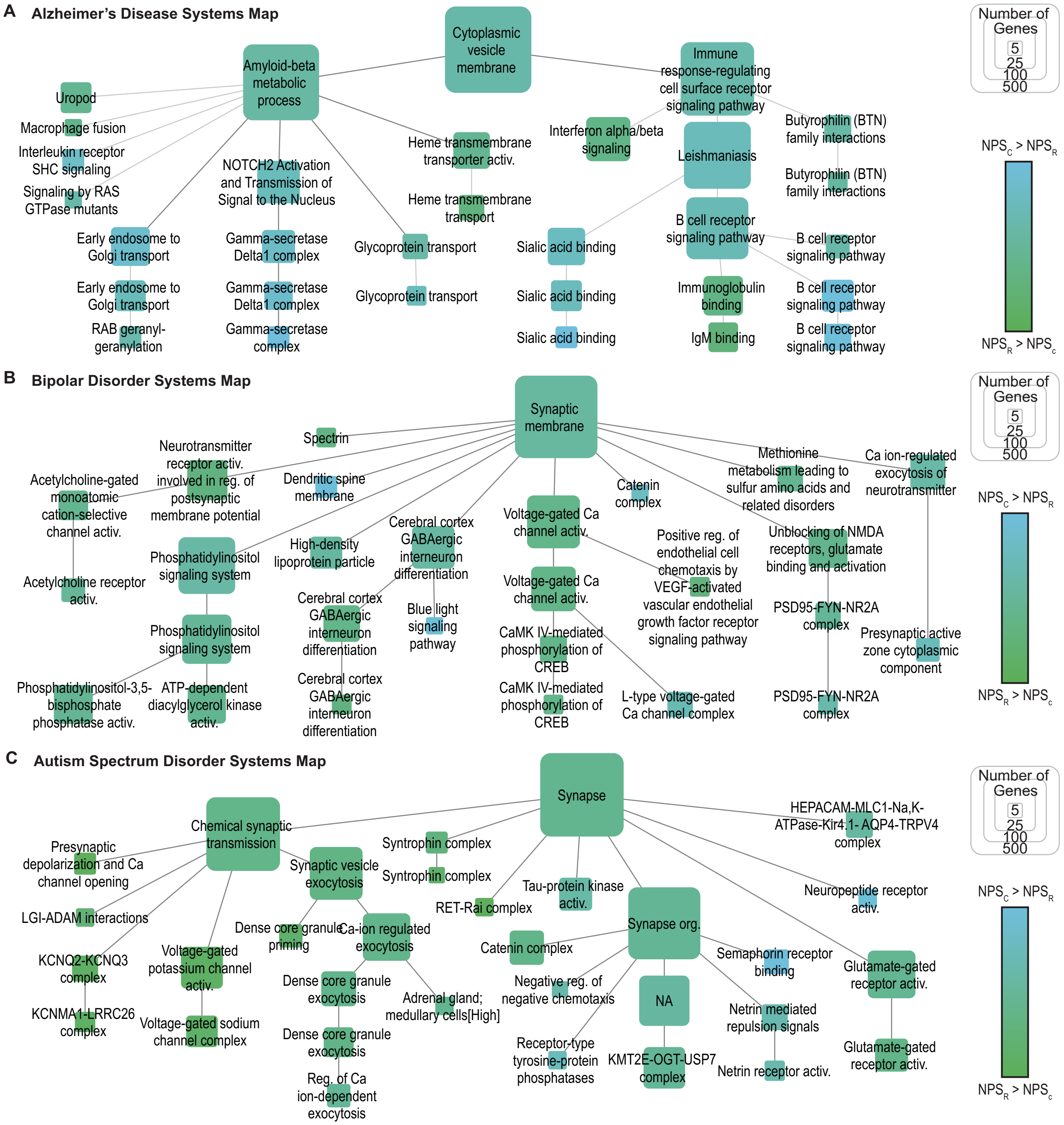

Figure EV5. Systems maps constructed from the trait-specific networks for select neuropsychiatric traits.

Maps generated via hierarchical community detection of the trait-specific networks for (**A**) Alzheimer's disease, (**B**) bipolar disorder, and (**C**) autism spectrum disorder, and annotated using g:Profiler (Methods). Node size indicates the number of genes per system, and node color represents the ratio of NPS_C and NPS_R for genes within the system. Systems with more than five genes and that could be annotated using g:Profiler are displayed. Underlying trait-specific networks were filtered to genes expressed in at least one brain region with TPM > 1 (HPA). Related to Fig. 6.
